# Intrinsic Phonons as a Dynamic Control Knob for Catalytic Reactivity in 2D Materials

**DOI:** 10.1002/advs.76843

**Published:** 2026-07-27

**Authors:** Kai Ren, Feifan Wang, Yong‐Wei Zhang, Tianyang Liu, Liangzhi Kou, Yu Jing

**Affiliations:** ^1^ School of Mechanical and Electronic Engineering Nanjing Forestry University Nanjing China; ^2^ Co‐Innovation Centre of Efficient Processing and Utilization of Forest Resources College of Chemical Engineering Nanjing Forestry University Nanjing China; ^3^ Institute of High Performance Computing A*STAR Singapore Singapore; ^4^ School of Mechanical Medical and Process Engineering Queensland University of Technology Brisbane Queensland Australia

**Keywords:** first‐principles simulations, Janus transition metal dichalcogenides, lattice vibration, oxygen evolution reaction, phonon engineering

## Abstract

Catalytic activity is conventionally understood in terms of static electronic structure descriptors, with lattice vibrations treated as a passive background. Here we show that intrinsic phonon modes can serve as an active and selective control knob for catalytic reactivity in 2D materials. Using density functional theory, we demonstrate that mode‐specific lattice excitations in transition metal dichalcogenides dynamically modulate their electronic structure, inducing direct–indirect bandgap transitions and substantial bandgap renormalization. In Janus WSSe, excitation of the *A*1 2 mode reduces the bandgap to 0.93 eV, significantly enhancing carrier transport. More importantly, these phonon‐induced lattice distortions systematically tune adsorption energetics and reaction pathways. Using the oxygen evolution reaction as a model system, we find that phonon activation lowers the overpotential by up to 17%, arising from weakened adsorption of key intermediates. Crystal orbital Hamilton population and *p*‐band center analyses reveal that this effect originates from phonon‐driven modulation of orbital hybridization and bonding strength. Our results establish a dynamic, mode‐selective paradigm for controlling catalytic processes via intrinsic lattice degrees of freedom, opening a route toward phonon‐engineered electrocatalysis beyond static materials design.

## Graphical abstract

Intrinsic phonon excitations are demonstrated to serve as a dynamic control knob for catalytic reactivity in 2D materials. Mode‐selective lattice vibrations reconstruct the electronic structure, lower oxygen evolution reaction barriers, establishing a phonon‐engineering paradigm for dynamically regulating electrocatalysis beyond conventional static catalyst design.

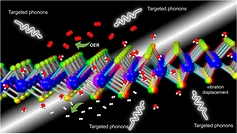

## 1 Introduction

Dynamic modulation of catalytic processes through external fields, such as light, electricity, and magnetic fields, has emerged as an effective strategy to enhance catalytic performance [1]. Among them, wave catalysis, which employs electromagnetic waves of specific frequencies to interact with catalysts and tune their reaction pathways, represents an exciting frontier [2]. A well‐established example is microwave catalysis [3], where external electromagnetic excitation enhances reactions through volumetric energy absorption and selective activation of polar or conductive materials, leading to faster kinetics and improved selectivity [4, 5]. For instance, low‐frequency vibration‐assisted catalysis promotes bond activation and enhances mass transport in heavy‐oil aquathermolysis [6]. Unlike conventional heating, microwave irradiation (typically 300 MHz–300 GHz) interacts with dipolar or ionic species, inducing rotational or vibrational motions that create localized “hot spots” and non‐thermal activation phenomena [7]. Emerging evidence suggests that these effects extend beyond macroscopic heating [8]. Similarly, high‐frequency electromagnetic excitations, such as lasers or optical irradiation, can modulate catalysis [9, 10] by coupling to electronic or plasmonic transitions [11, 12]. For example, photothermal catalysis couples photon absorption with lattice vibrations to accelerate reactions [13], while photon–phonon cascade catalysis enables efficient methane‐to‐formaldehyde conversion under mild conditions [14]. Even in enzyme catalysis, femtosecond–picosecond vibrational motions can transiently modulate reaction barriers, underscoring the universal role of ultrafast dynamics in catalytic processes [15]. All these evidences point to a unifying picture in which lattice and molecular vibrations play a central role in mediating energy transfer and reaction pathways across diverse catalytic systems, suggesting phonons can act as an active control knob for catalytic reactivity.

Recent studies have further extended these concepts to photon–phonon and vibration‐assisted catalysis, where both photonic and phononic excitations jointly regulate catalytic activity and selectivity [16]. Across the electromagnetic spectrum, from microwaves to lasers, external fields can reshape reaction energetics through interactions with molecular or lattice dynamics [14, 15, 16]. Such phenomena are often attributed to frequency matching, where the microwave frequency resonates with molecular or lattice relaxation modes, selectively exciting specific degrees of freedom and lowering the activation barrier. These observations highlight the potential of frequency‐dependent energy input to manipulate catalytic reactions at the microscopic level [2]. Despite these advances, the wave frequency mechanisms underlying frequency‐dependent catalytic enhancement remain elusive. It is unclear whether external fields directly excite intrinsic vibrational (phonon) modes in catalysts, and to what extent such resonances modify electronic structure or reaction energetics. Therefore, we will demonstrate that intrinsic phonon modes act as an internal, reversible, and mode‐selective tuning knob for catalytic reactivity. Here, we emphasize that the proposed mechanism is intrinsically mode‐selective rather than thermal in nature. Unlike thermal excitation, which involves a broad distribution of phonon modes, our approach targets specific vibrational eigenmodes that induce well‐defined lattice distortions and selective modulation of electronic structure and adsorption energetics. It is worth emphasizing that when an intense terahertz laser pulse interacts with a material, it can selectively excite specific lattice vibrational modes, namely infrared‐active phonons. Through nonlinear phonon coupling, these driven modes can induce transient lattice distortions, thereby modifying the crystal symmetry of the material. This approach, commonly referred to as nonlinear phononics, has emerged as a powerful strategy for dynamically controlling quantum states in condensed matter systems [17]. Besides, such mode‐selective excitation is experimentally accessible via techniques such as ultrafast laser and terahertz driving [18, 19, 20]. For example, terahertz excitation pulses have been utilized to directly drive optical phonon modes in MAPbI_3_, inducing significant perturbations in electronic relaxation dynamics and thereby highlighting the pivotal role of phonons in governing charge‐carrier behavior [21]. Similarly, the selective excitation of molecular vibrational modes has been shown to effectively modulate device performance in organic optoelectronic systems [22]. Extensive theoretical and experimental investigations have systematically elucidated nonequilibrium carrier–phonon interactions under photoexcitation. These processes encompass the quantum emission of longitudinal optical (LO) phonons, the subsequent cascade decay of optical phonons into acoustic modes, as well as the emergence of multichannel relaxation pathways under conditions of high carrier injection [23]. Therefore, the proposed phonon‐mediated modulation represents a realistic and distinct pathway for controlling catalytic reactivity.

Furthermore, 2D materials are selected as model systems to validate the proposed concept of phonon‐mediated catalytic modulation. This is because 2D materials possess reduced dimensionality, well‐defined lattice structures, and strong coupling between lattice vibrations and electronic states, making them particularly suitable for resolving mode‐specific phonon effects. Within this category, transition metal dichalcogenides (TMDs) are further chosen due to their tunable electronic structures, rich phonon spectra, and established catalytic relevance [24]. In addition, the oxygen evolution reaction (OER) is employed as a model reaction because it involves complex multi‐step proton–electron transfer processes [25] and is highly sensitive to variations in electronic structure and adsorption energetics, making it an ideal probe for evaluating phonon‐induced catalytic modulation [26]. Therefore, the selected material–reaction combination serves as a representative platform to demonstrate the generality of the proposed framework.

In this work, we first develop a phonon‐mode‐resolved first‐principles framework to directly quantify how intrinsic lattice vibrations modulate catalytic reactivity in TMDs. By explicitly introducing atomic displacements corresponding to individual phonon eigenmodes, we establish a dynamic structure–reactivity relationship that bridges phonon frequencies with electronic reconstruction and oxygen evolution energetics. The simulations uncover that specific high‐frequency modes, particularly the *A*2 1 vibration in the WSSe monolayer, markedly reduce the OER overpotential by weakening intermediate adsorption and narrowing the bandgap. These results provide concrete microscopic evidence that intrinsic phonon excitations can serve as a dynamic control knob for catalytic enhancement, offering a new theoretical route for the rational design of frequency‐responsive and phonon‐regulated electrocatalysts.

## 2 Computational Methods

### 2.1 Phonon‐Mode‐Resolved Lattice Perturbation Approach

We propose a mode‐resolved phonon engineering framework to explicitly elucidate the role of lattice vibrations in catalytic processes, as illustrated in Figure 1. Instead of treating phonons merely as thermal perturbations, we explicitly account for lattice dynamics by selectively activating specific Γ‐point vibrational eigenmodes. The choice of Γ‐point phonons is further motivated by both theoretical and experimental considerations. From the theoretical perspective, Γ‐point (*q* = 0) optical phonons correspond to long‐wavelength collective lattice vibrations that preserve the translational periodicity of the primitive cell. Consequently, the associated frozen‐phonon configurations maintain crystal symmetry and enable direct evaluation of electron–phonon coupling without introducing additional momentum‐dependent scattering, providing an ideal framework for establishing a clear structure–property relationship. Within the Allen–Heine–Cardona formalism, Γ‐point frozen‐phonon calculations have been widely employed to evaluate phonon‐induced band renormalization and lattice‐driven electronic reconstruction. Following a frozen‐phonon scheme, selected Γ‐point phonon eigenmodes were projected onto the equilibrium lattice, where atomic displacements were applied along the corresponding eigenvectors with carefully controlled amplitudes. This procedure generates symmetry‐preserved, phonon‐activated configurations that capture mode‐specific lattice distortions without introducing artificial momentum scattering. Owing to the translational symmetry retained at the Γ point, phonon‐induced effects can be cleanly isolated, enabling a rigorous analysis of individual vibrational contributions.

**FIGURE 1:**
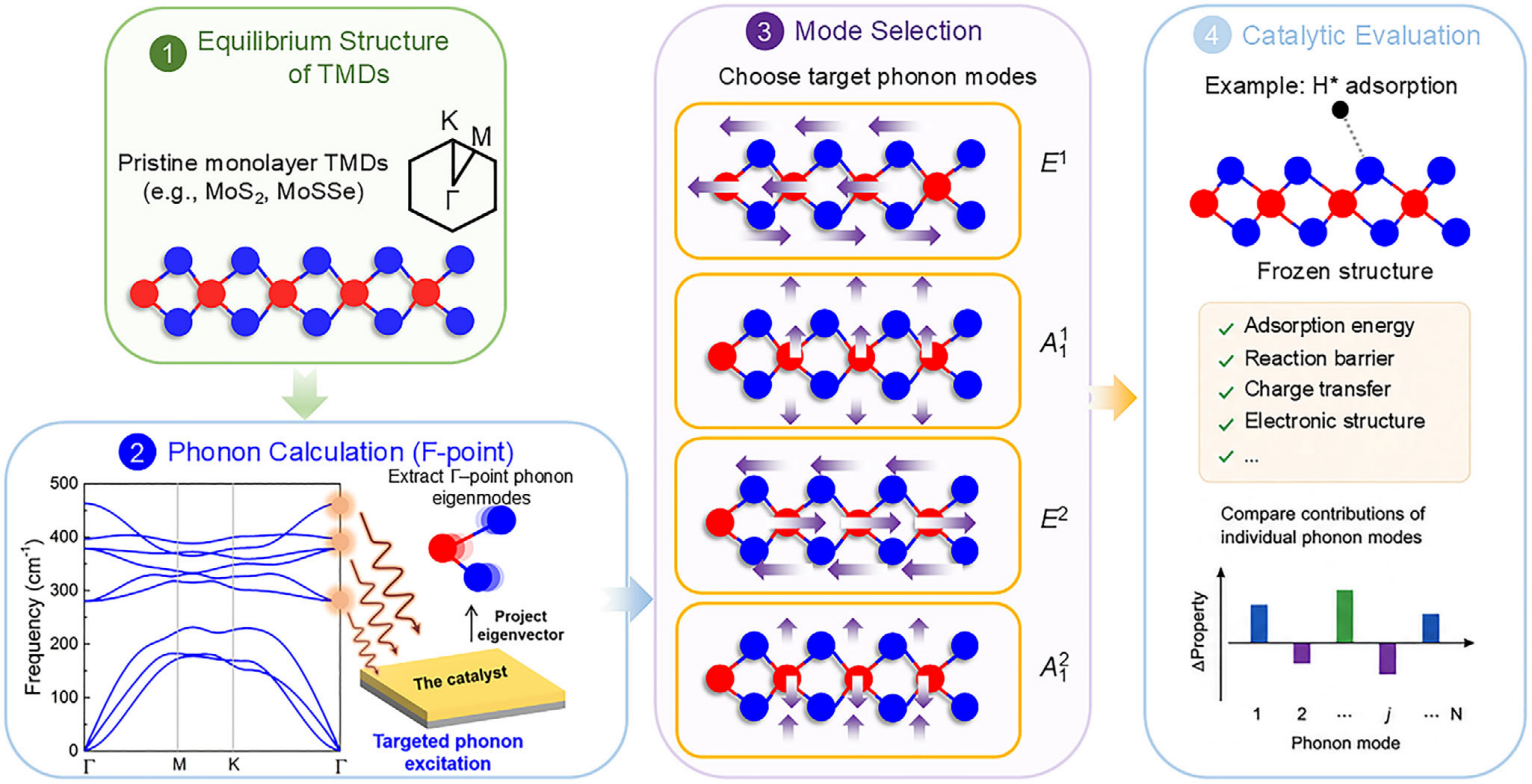
Schematic illustration of the frozen‐phonon approach combined with mode‐resolved catalytic analysis. Selected Γ‐point phonon eigenmodes are projected onto the equilibrium structure to generate phonon‐activated configurations, which are subsequently used for DFT evaluation of electronic and catalytic properties.

It should be emphasized that the frozen‐phonon configurations considered in this work should not be interpreted as arbitrary static lattice distortions. Instead, they represent instantaneous snapshots of atoms displaced strictly along the eigenvectors of specific phonon modes. Within the harmonic approximation and the Born–Oppenheimer framework, such frozen configurations provide the static limit of mode‐selective lattice vibrations and constitute a well‐established approach for evaluating electron–phonon coupling and phonon‐induced electronic structure renormalization [27, 28]. Therefore, the observed changes in adsorption energetics originate from phonon‐mode‐specific lattice displacements coupled with the corresponding electronic response, rather than from unconstrained geometric perturbations [29, 30].

### 2.2 Density Functional Theory

All first‐principles calculations in this investigation are based on density functional theory (DFT) using the Vienna ab initio simulation package (VASP) [31, 32, 33]. Besides, the electron exchange and correlation are described using the Perdew–Burke–Ernzerhof (PBE) method embedded with the generalized gradient approximation (GGA) functional [34, 35, 36, 37]. Then, the hybrid Heyd–Scuseria–Eenzerhof (HSE06) functional is applied to correct the underestimated electronic properties of the studied system [38]. The DFT‐D3 functional provided by Grimme is used to correct the interlayer weak van der Waals (vdWs) interactions in the heterostructure [39]. Monkhorst–Pack *k*‐point grids of 15 × 15 × 1 and 17 × 17 × 1 are used for the relaxation and self‐consistent simulations, respectively. The vacuum space of 25 Å is considered to prevent the interaction of the nearby layers. The force and energy convergence are controlled by 0.01 eV·Å^−1^ and 0.01 me·V, respectively. In addition, the phonon dispersion is obtained by the PHONOPY code [29, 40] from the density functional perturbation theory.

### 2.3 Harmonic Lattice Dynamics Framework

It is considered that the potential energy of the TMD crystal, when atoms undergo slight deviations from their equilibrium sites, can be expressed as a power expansion in terms of the nuclear displacements (*u*) as Equations S1–S3. The derivatives are determined at the atoms’ equilibrium sites within the crystal lattice, corresponding to absolute zero and neglecting zero‐point energy. Here, *lk* denotes the *k*‐th atomic species (*k* = 1, 2, …, *n*) located in the *l*‐th unit cell. By adopting this form, the adiabatic approximation is invoked: electrons are assumed to adjust instantly to the nuclear displacements, and the resulting electronic energy variation in the distorted lattice is incorporated into an effective potential between nuclei. And the extra terms in Equation S1 are implicitly included by evaluating the second derivatives at the average atomic positions corresponding to temperature *T*:

(1) $$\varphi_{xy}\left(l\kappa ,l^{\prime }\kappa^{\prime }\right)=\frac{\partial^{2}\varphi }{\partial x\left(l\kappa \right)\partial y\left(l^{\prime }\kappa^{\prime }\right)}$$

For the *k*‐th atom in the *l*‐th unit cell, the motion equation under the harmonic force assumption is expressed as:

(2) $$m_{k}\frac{\partial^{2}}{\partial t^{2}}\left[u_{x}\left(l\kappa \right)\right]=-\frac{\partial \varphi_{2}}{\partial u_{x}\left(l\kappa \right)}=-\sum_{l^{\prime }k^{\prime }y}\varphi_{xy}\left(l\kappa ,l^{\prime }\kappa^{\prime }\right)u_{y}\left(l^{\prime }\kappa^{\prime }\right)$$

It is assumed that the displacements can be expressed as a combination of propagating waves; the displacements can be further obtained as:

(3) $$u_{x}\left(l\kappa \right)=U_{x}\left(\kappa q\right)\exp i\left[q.r\left(l\kappa \right)-\omega \left(q\right)t\right]$$

The general solution is obtained by summing the right‐hand side over all permissible values of *q*, the wave vector. The coordinate of the *l*‐th unit cell is denoted as *r*(*l*), while the equilibrium position of the *k*‐th atom within this cell is expressed as *r*(*lk*) = *r*(*l*) + *r*(*k*). Equation (3) can equivalently be written using *r*(*l*) instead of *r*(*lk*), which only shifts the phase of *U_x_* (*kq*). Substituting Equation (3) into Equation (2), we can obtain:

(4) $$m_{\kappa }\omega^{2}\left(q\right)U_{x}\left(\kappa q\right)=\sum_{\kappa^{\prime }y}M_{xy}\left(\kappa \kappa^{\prime },q\right)U_{y}\left(\kappa^{\prime }q\right)$$

By inserting a specific frequency *ω_j_* (*q*) into Equation (4), one obtains a series of quantities *U_xj_* (*kq*). These form the components of a column vector *U_j_* (*q*), which characterizes the displacement pattern of atoms associated with that vibrational mode. The wave amplitude defined in Equation (3) can then be expressed as:

(5) $$U_{x}\left(\kappa q\right)=\sum_{j}U_{xj}\left(\kappa q\right)$$

For the TMD heterostructure with periodic boundary conditions, the wave amplitudes can be denoted as:

(6) $$u_{x}\left(l\kappa \right)=U_{x}\left(\kappa q\right)\exp\left\{i\left[q.r\left(l\right)-\omega \left(q\right)t\right]\right\}$$

which possesses the periodicity of *w*(*q*). However, they are less practical because they produce complex matrix elements *M_xy_* (*kk’*) even when all atoms are located at symmetry centers. The potential energy of the crystal expressed in terms of the normal modes is found to be:

(7) $$E_{j}\left(q\right)=mN{\left|A_{j}\left(q\right)\right|}^{2}{\omega_{j}}^{2}\left(q\right)=\left[n_{j}\left(q\right)+\frac{1}{2}\right]\hbar \omega_{j}\left(q\right)$$

Since the oscillator energy states are quantized, *n_j_* (*q*) represents the mode occupation number, such as the phonon count. In a crystal at thermal equilibrium with temperature *T*, this relation reflects the underlying symmetry:

(8) $$n_{j}={\left[\exp\left(\hbar \omega_{j}/k_{B}T\right)-1\right]}^{-1}$$

## 3 Results and Discussion

Considering the unique Janus interface, the MoSSe and WSSe monolayers are selected, and the optimized lattice constants are calculated as 3.228 and 3.269 Å, respectively, agreeing well with previous reports [41, 42]. The projected band structures of MoSSe and WSSe monolayers are calculated and shown in Figure 2a,b, respectively. One can see that the direct bandgap is obtained as 2.106 and 2.075 eV for MoSSe and WSSe monolayers, respectively, at the HSE06 level with the CBM and VBM at the *K*‐point. Obviously, the CBM and VBM of MoSSe and WSSe monolayers are dominated by Mo or W atoms, respectively. The calculated phonon spectra of the MoSSe and WSSe monolayers are further provided in Figure 2c,d, respectively, with the maximal frequency of about 437.84 and 402.87 cm^−1^. Since the unit cells of MoSSe and WSSe monolayers both include three atoms, there are nine phonon modes (three acoustic modes and six optical modes). Using the factor group investigation of the point group, we can obtain six long‐wavelength optical phonon modes at the Г point, which can be expressed by:

(9) $$\begin{array}{ccc} \Gamma_{\mathrm{optical}}\left(\mathrm{MoSSe}\;\mathrm{or}\;\mathrm{WSSe}\right) & = & E^{1}\left(R\right)+A_{1}^{1}\left(\mathrm{IR}+R\right)+E^{2}\left(R\right)\\ & & +A_{1}^{2}\left(\mathrm{IR}+R\right) \end{array}$$

where all the R and IR represent the Raman and infrared modes, respectively. One can see that the MoSSe and WSSe monolayers show similar phonon properties, in which two of the optical branches are degenerate, and two are non‐degenerate at the Г point. While the degenerate and non‐degenerate points are in a staggered arrangement. Furthermore, the atomic vibration modes with different phonon frequencies at the Г point of MoSSe and WSSe monolayers are demonstrated in Figure 2e,f, respectively. And the calculated corresponding displacement of lattice vibrations of the MoSSe and WSSe monolayers under different intrinsic phonon modes are summarized in Tables S1 and S2. These excited vibration displacements correspond to sub‐ångström lattice distortions. Such amplitudes are sufficiently small to preserve the harmonic character of the lattice while remaining comparable to experimentally reported coherent optical phonon displacements generated by resonant terahertz or mid‐infrared excitation [43, 44]. Therefore, the adopted frozen‐phonon configurations represent physically realistic snapshots of nonequilibrium lattice vibrations that are experimentally accessible under current ultrafast excitation techniques. The lattice vibration displacements in MoSSe and WSSe monolayers are in the horizontal direction for the Raman mode, while the vertical atomic displacements are addressed in the mode assembly with infrared and Raman modes, which also describes the vibration direction and displacement under intrinsic phonon frequency excitation.

**FIGURE 2:**
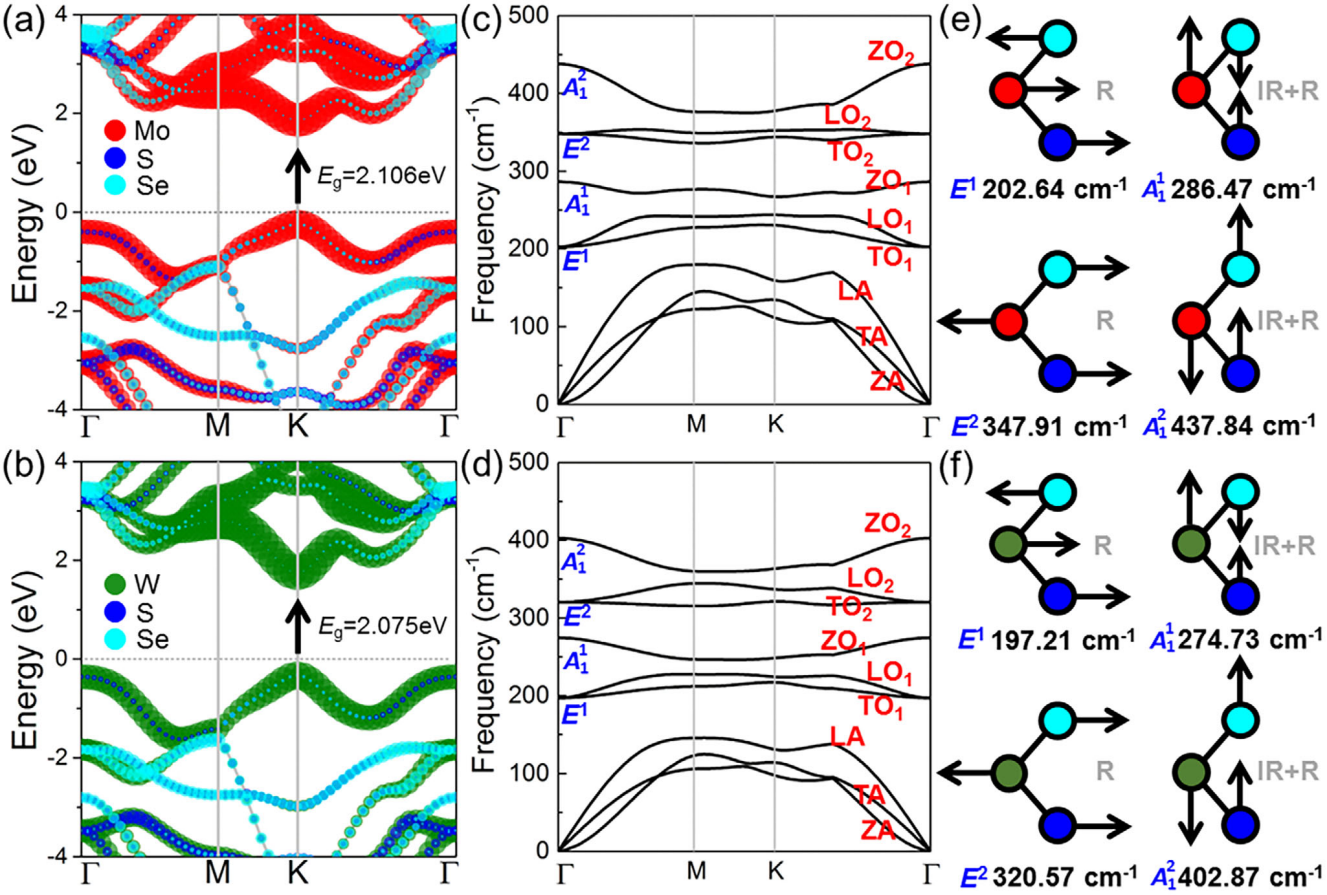
(a,b) The projected band structures, (c,d) phonon spectra and (e,f) lattice vibration modes of (a,c,e) MoSSe and (b,d,e) WSSe monolayers. The Fermi level is marked as 0 eV. Blue, green, cyan and red spheres represent the S, W, Se and Mo atoms, respectively.

When we include a phonon degree of freedom (frequency, *ω*_ph_​) into the Hamiltonian of the MoSSe and WSSe systems, the form of the Schrödinger equation itself does not change, which is still expressed by Equation S4. For a single phonon mode with frequency *ω*_ph_​, the phonon Hamiltonian (*Ĥ*_ph_) is demonstrated as:

(10) $${\hat{H}}_{\mathrm{ph}}=h\omega \mathrm{ph}\left(b†b+1/2\right)$$

where *b* ^†^ and *b* are phonon creation and annihilation operators. Thus, the total Hamiltonian becomes:

(11) $$\hat{H}={\hat{H}}_{e}+{\hat{H}}_{\mathrm{ph}}+{\hat{H}}_{e-\mathrm{ph}}$$

where the last term represents the electron–phonon interaction. A common model is the Holstein‐type coupling with coupling constant g as follows:

(12) $${\hat{H}}_{e-\mathrm{ph}}=g\sum ci†ci\left(b†\;+b\right)$$

The Schrödinger equation is still [45]:

(13) $$ih\partial \Psi \left(r,t\right)/\partial t=\left({\hat{H}}_{e}+h\omega \mathrm{ph}\left(b†b+1/2\right)+{\hat{H}}_{e-ph}\right)\Psi \left(r,\left\{n_{\mathrm{ph}}\right\},t\right)$$

where the wavefunction Ψ is now defined in a larger Hilbert space that includes both electronic states and phonon Fock states ∣*n*_ph_⟩. One can see that the influence of a given phonon frequency (*ω*_ph_​) on the Hamiltonian of Janus TMDs has been addressed. Such a phonon‐driven modification of the Hamiltonian further affects the total energy of the Janus TMD system, implying that lattice vibrations can play a significant role in tuning the electronic and thermodynamic properties. When the TMD materials are excited by the natural frequency of phonons, the Mo (or W) and S atoms in the lattice will also shift in their equilibrium positions. It is worth noting that this shift can be calculated. We express the change in the electronic eigenenergy *E*, of band *n* at wave vector *k*, arising from static deviations of atoms from their equilibrium positions, in terms of a second‐order Taylor series expansion [46]:

(14) $$\Delta E_{n,k}=u\cdot \nabla E_{n,k}+\frac{1}{2}u\cdot D\cdot u$$

where u denotes the displacement vector, while D represents the associated Hessian matrix. The displacements are conventionally expanded as a linear combination of normal modes [47]:

(15) $$u\left(\alpha ,\kappa \right)=\sum_{j}{\left(\frac{\hbar }{2M_{\kappa }\omega_{j}}\right)}^{1/2}\varepsilon_{j}\left(\alpha ,\kappa \right)\left(\alpha_{j}^{+}+a_{j}\right)$$

where u (*α*, *κ*) denotes the displacement of atom *κ* in the unit cell along direction *α*, where the atom has mass *M_κ_*. The electron−phonon interaction is determined within the frozen‐phonon framework. Consequently, the summation is restricted to zone‐center vibrational modes *j*​ with frequency *ω*_ph_. To ensure numerical accuracy, a sufficiently large supercell effectively providing dense Brillouin‐zone sampling is required for convergence. Here, *α*+ *j*, and *αj* represent the phonon creation and annihilation operators, while *ε* (*α*, *κ*) corresponds to the components of the normalized polarization vectors. By inserting Equation (15) into Equation (14) and taking the thermal average [47], we find that under the harmonic approximation the term linear in u disappears [28]:

(16) $$\Delta E_{n,k}=\sum_{j}\frac{\partial E_{n,k}}{\partial n_{j}}\left(n_{j}+\frac{1}{2}\right)$$

where *n_j_* = (*e^βhωj^* − 1)^−1^ denotes the Bose–Einstein distribution for phonon mode *j*, and the electron–phonon interaction coefficient *∂E_n_*_,_ *_k_*/*∂n_j_* is expressed as:

(17) $$\frac{\partial E_{n,k}}{\partial n_{j}}=\frac{1}{2}x_{j}\cdot D\cdot x_{j}$$

where *x_j_* (*α*, *κ*) denotes the frozen‐phonon displacement. According to Equation (13), this corresponds to the quadratic component of Δ*E_n_*_,_ *_k_* when atoms are displaced along a given frozen‐phonon mode *x_j_* ​. In practice, ∂*E_n_*_,_ *_k_*/∂*n_j_* is obtained by carrying out electronic structure calculations for the chosen *x_j_* ​ and averaging the resulting energy variations, which effectively cancels the linear contribution. Thus, we calculate the band structure after loading the atomic vibration displacement excited by phonons to the equilibrium position of the MoSSe and WSSe monolayers to examine the effect of intrinsic lattice vibration on the band structure of these Janus TMD monolayers.

To investigate the effects of phonon excitation on the electronic performance of MoSSe and WSSe monolayers, the vibration displacement of four vibration modes is projected into the optimized structure as a vector. The projected band structures of the MoSSe and WSSe monolayers excited by the phonon mode at the Г point are obtained as Figure 3a,b, respectively. Clearly, the MoSSe and WSSe monolayers still retain the semiconducting nature; the conduction band minimum (CBM) and valence band maximum (VBM) come from Mo or W atoms excited by these lattice vibration modes. The bandgap of MoSSe (WSSe) monolayer is tuned to 2.091 eV (2.063 eV), 1.895 eV (1.721 eV), 2.071 eV (2.033 eV), and 2.011 eV (0.934 eV), respectively, under the *E* ^1^(R), *A*1 1 (IR+R), *E* ^2^(R) and *A*2 1 modes. Compared with the bandgap of the ground state, the MoSSe monolayer presents a larger bandgap induced by the lattice vibration mode, as shown in Figure 3a. The WSSe monolayer shows a comparable bandgap under the *E* ^1^ and *E* ^2^ modes, and the bandgap can be reduced by the *A*1 1 and *A*2 1 modes, as demonstrated by Figure 3b. In particular, the pronounced decrease in bandgap of 0.934 eV is observed in the WSSe monolayer by the *A*2 1 mode, as suggested in Figure 2f. Such a decent bandgap implies changes in catalytic activity. Although such excited bandgap narrowing is beneficial but is not the primary descriptor governing the OER activity. The dominant origin is the phonon‐induced modulation of adsorption energetics through orbital hybridization, which is discussed below.

**FIGURE 3:**
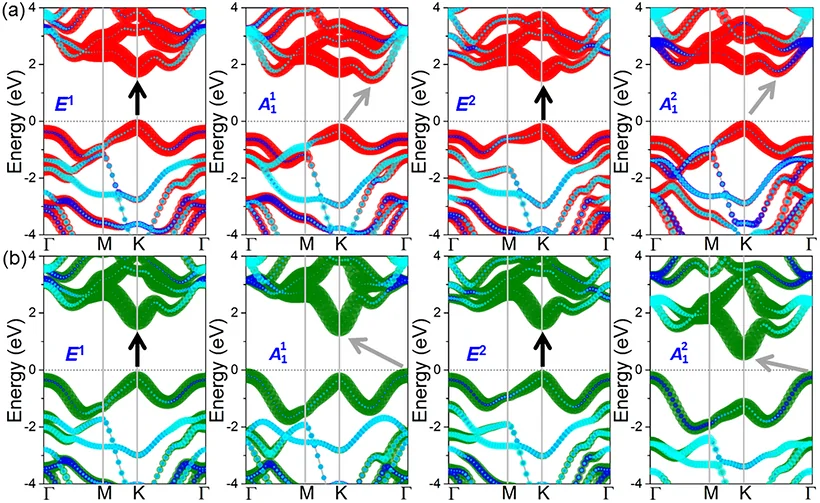
The projected band structures of Janus (a) MoSSe and (b) WSSe monolayers excited by the lattice vibration modes with different phonon frequencies at the Г point. The Fermi level is marked as 0 eV. Blue, green, cyan, and red spheres represent the S, W, Se, and Mo atoms, respectively.

To verify the universality of the effects of lattice vibrations in different phonon modes on the electronic properties of TMD materials, we further calculated the electronic band structures of representative TMDs (MoS_2_ and WS_2_ monolayers) under phonon‐excited states. We calculated the phonon spectra and lattice vibration characteristics of the MoS_2_ and WS_2_ monolayers, as shown in Figure S1. The long‐wavelength optical phonon modes at the Γ point can be decomposed as in Equation S5. The obtained frequencies for these modes, demonstrated in Figure S1b,d, which is good agreement with experimental reports [48, 49]. The projected band structure of MoS_2_ (or WS_2_) monolayer at the ground state is presented in Figure S2a (or c), which shows a direct bandgap of about 2.147 and 2.344 eV, respectively, and the CBM and VBM of MoS_2_ (or WS_2_) monolayer mainly result from the Mo (or W) atom, agreeing well with other reports [50]. Figure S2b (or d) demonstrates that phonon engineering can also effectively influence the electronic properties of MoS_2_ (or WS_2_) monolayers. In particular, the vibration modes of *A*1 1 and *A*2 2 are the most effective. The projected band structure of MoS_2_ and WS_2_ monolayers excited by different vibration modes is shown in Figure S3a,b, respectively. One can see that the CBM and VBM of the MoS_2_ (or WS_2_) monolayer still result from Mo (or W) atom, while the bandgap is redefined as 2.105 eV (or 2.302 eV), 2.121 eV (or 2.316 eV), 1.223 eV (or 2.083), and 1.682 eV (or 2.045 eV), respectively, excited by the intrinsic *E* ^2^, *E* ^1^, *A*1 1 and *A*2 2 modes. It is obvious that the most sensitive band structure of the MoS_2_ (or WS_2_) monolayer is excited by non‐degenerate phonon modes (*A*1 1 and *A*2 2 modes), decreased by about 40% (or 13%) compared with that of the ground state, which is also consistent with the fact that excitation of non‐degenerate states exerts the most pronounced influence on the energy levels of Janus TMDs.

More interestingly, the transformation between the direct and indirect bandgaps in MoSSe and WSSe monolayers is obtained. As shown in Figure 4a,b, the direct bandgap can be obtained by the ground state and *E* (*E* ^1^ and *E* ^2^) mode, while the indirect bandgap is excited by *A* (*A*1 1 and *A*2 1) mode in MoSSe and WSSe monolayers, respectively. Excitingly, similar to the Janus TMDs system, the direct (or indirect) bandgap can also be addressed in MoS_2_ (or WS_2_) monolayer under degenerate (or nondegenerate) state modes of the intrinsic phonon, as demonstrated in Figure S4. Besides, even though the band structures of MoSSe and WSSe monolayers can be manipulated by different lattice vibration modes, the band edge positions still present a decent potential to promote the oxidation evolution reaction for water splitting at pH 0, shown in Figure 4a,b, suggesting tunable photocatalytic OER performance by phonon engineering. For example, the calculated CBM (or VBM) edge energy of the WSSe monolayer under ground state, intrinsic *E* ^1^, *A*1 1, *E* ^2^ and *A*2 2 modes are −4.120 eV (−6.195 eV), −4.133 eV (−6.196 eV), −4.370 eV (−6.091 eV), −4.121 eV (−6.155 eV) and −4.356 eV (−5.690 eV), which is desirable to promote water oxidation (−5.67 eV) and reduction (−4.44 eV) at pH 0 [51]. All these results indicate that the proposed lattice vibration modulation exhibits a certain degree of universality in governing the electronic properties of TMD materials.

**FIGURE 4:**
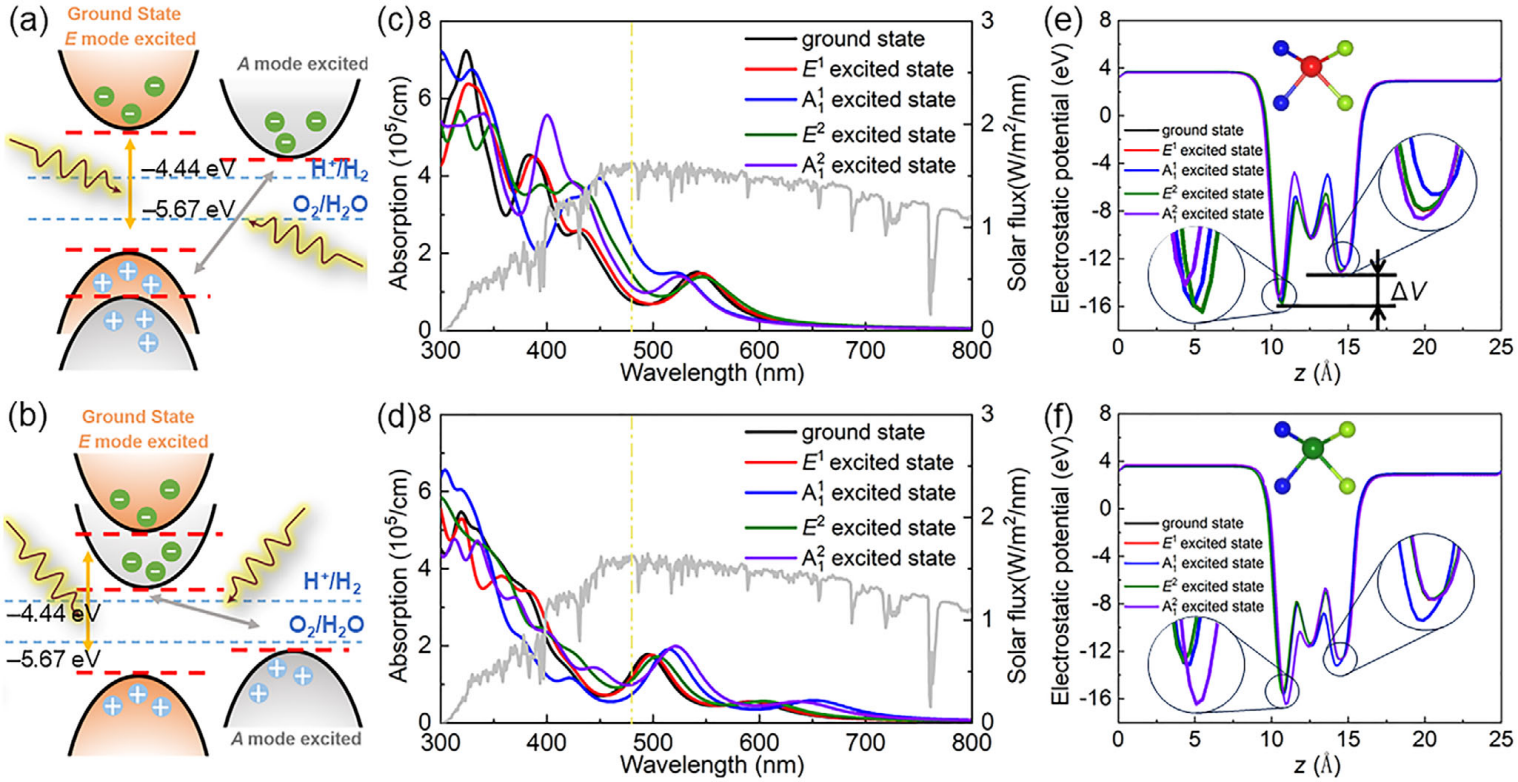
The band edge positions of the Janus (a) MoSSe and (b) WSSe monolayers excited by the lattice vibration modes with different phonon frequencies at the Г point, compared with the redox potential of the water splitting at pH 0. The calculated transient (c,d) light absorption characteristics and (e,f) potential drop (Δ*V*) vertical across the sandwich structure direction of the (c,e) MoSSe and (d,f) WSSe monolayers.

The transient light absorption of the Janus MoSSe and WSSe monolayers is further investigated by Equations S6 and S7. The obtained light absorption spectrum of these Janus TMDs under ground state and different excited states is further studied as demonstrated in Figure 4c,d, respectively. One can see that the transient light absorption performance of the MoSSe (or WSSe) monolayer can be obviously enhanced by non‐degenerate phonon‐excited states (*A*1 1 mode) with the absorption peak of 5.58 × 10^5^ cm^−1^ (or 1.89 × 10^5^ cm^−1^) at a wavelength of 400 nm (or 514 nm). By the *A*2 1 excited mode, the absorption peak of the MoSSe (or WSSe) is improved to 1.39 × 10^5^ cm^−1^ (or 2.04 × 10^5^ cm^−1^) at a wavelength of 525 nm (or 524 nm). The asymmetry of the Janus structure endows MoSSe and WSS with particular built‐in electric fields as potential power for photoexcited charge carriers. Thus, the phonon‐excited potential drop across the MoSSe and WSS is further calculated in Figure 4e,f, respectively. The maximal potential of the MoSSe (or WSSe) monolayer is obtained as 3.045 eV (or 3.802 eV) by the *A*2 1 (or *A*2 1) excited mode.

Coincidentally, these non‐degenerate phonon modes (*A*1 1 and *A*2 1) can lead to the transformation of MoSSe and WSSe into indirect bandgap semiconductors; such an indirect bandgap can suppress the recombination of photogenerated electrons and holes, thereby prolonging the lifetime of photogenerated carriers. Besides, the non‐degenerate phonon mode can also enhance the transient light absorption and interface driving force of the MoSSe and WSSe monolayers for photogenerated carriers; thus, non‐degenerate phonon excitation endows MoSSe and WSSe with superior photocatalytic performance compared to their ground states. Therefore, we further investigate the Gibbs free energy characteristics of the system under such excited states.

Building on our investigation of the influence of lattice vibrations on the electronic band structures of MoSSe and WSSe monolayers, it is natural to extend this analysis to their photocatalytic behavior. Since lattice vibrations can strongly couple with charge carriers and modify the density of states near the Fermi level, they are expected to play a significant role in governing the adsorption energetics and charge‐transfer processes involved in the OER. Therefore, exploring the impact of phonon dynamics on the OER performance of MoSSe and WSSe monolayers can provide deeper insight into the fundamental mechanisms underlying their catalytic activity. Accordingly, the Gibbs free energies (*G*) of MoSSe and WSSe monolayers under different lattice vibration modes during the OER process are examined by *G* = Δ*E* + Δ*E*_ZPE_–*T*Δ*S*, where Δ*E* presents the adsorption energy of the intermediates [52]. The overall reaction of the OER is expressed by Equations S8–S13, while Equations S14–S18 present the binding energies of different adsorbed systems for the intermediates in the Supporting Information. Δ*E*_ZPE_ represents the difference in zero‐point vibrational energy. *T* denotes the temperature (298.15 K in this study), while Δ*S* refers to the entropy change of the adsorbed intermediates on the MoSSe and WSSe monolayers under a standard pressure of approximately 101.325 kPa. For both the ground state and each phonon‐induced MoSSe and WSSe structure, the adsorption configurations in OER are independently optimized, and the lowest‐energy adsorption configuration is adopted for the subsequent OER free energy analysis. Then, the Gibbs free energies, calculated by Equations S19–S23, of the adsorbed intermediates (OH^*^, O^*^, and O^*^+OH^*^) on MoSSe and WSSe monolayers under various lattice vibration modes are presented in Figure 6a,b, respectively. One can see that the overpotential of the rate‐determining step of MoSSe and WSSe monolayers in the OER is the first reaction, at about 1.235 and 1.305 eV, respectively, under the ground state. Besides, the potential of Gibbs free energy in each step of the OER is slightly changed by lattice vibrations at different frequencies for the MoSSe monolayer, as shown in Figure 5a. The rate‐determining step of the MoSSe monolayer in the OER still remains the first step excited by different intrinsic lattice vibrations, which can be decreased to 2.345 eV under the *A*1 1 mode. Interestingly, compared with that of the MoSSe monolayer, the OER Gibbs free energy of the WSSe monolayer presents a more pronounced dependence on the lattice vibration regulation. From Figure 5b, even under different lattice vibration modes, the rate‐determining step of the OER is still the first step in the WSSe monolayer. In particular, under the high‐frequency excitation of *A*2 1 mode, the overpotential of the rate‐determining step of the OER is decreased to as low as 0.865 eV, reducing by 17% from that at the ground state. To further evaluate the possible influence of competitive adsorption during the OER, the adsorption energies of H_2_O^*^, OH^*^, and O^*^ were additionally calculated for the representative phonon‐excited systems by the *A*1 1 mode of MoSSe and the *A*2 1 mode of WSSe, which exhibit the largest reduction in OER overpotential. As shown in Figure S5, H_2_O adsorption is energetically less favorable than that of the oxygen‐containing intermediates, whereas OH^*^ and O^*^ remain preferentially adsorbed on the catalytic surface. Similar adsorption trends are observed for both phonon‐excited systems, indicating that competitive adsorption does not change the preferred adsorption behavior or the phonon‐induced catalytic enhancement mechanism proposed in this work. We also check the phonon‐engineering‐tunable OER performance of the MoS_2_ and WS_2_ monolayers, as demonstrated in Figure S6. The results demonstrate that both theory and simulation are sufficient to explain the feasibility of phonon engineering in controlling the OER performance of Janus TMDs (MoSSe and WSSe) and traditional TMDs (MoS_2_ and WS_2_) monolayers. It should be emphasized that bandgap narrowing and OER enhancement play different roles. While the reduced bandgap (such as WSSe with *A*2 1 mode) mainly facilitates charge transport by improving carrier excitation and conductivity, the intrinsic OER activity is primarily governed by the adsorption energetics of reaction intermediates. Thus, the *p*‐band center and orbital coupling are further explored.

**FIGURE 5:**
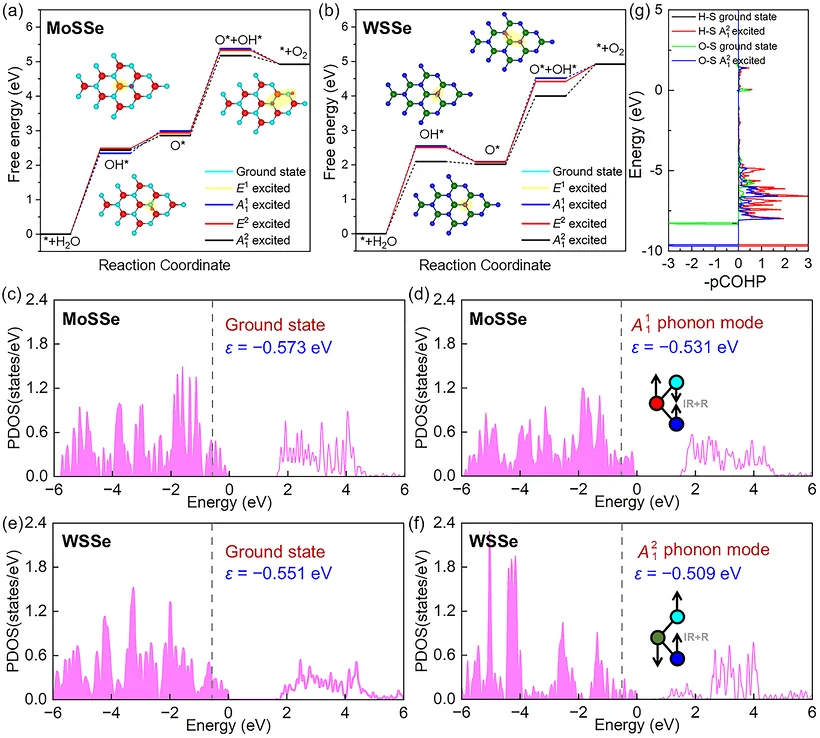
The calculated Gibbs free energy of the adsorbed intermediates in the OER on (a) MoSSe and (b) WSSe monolayers with the ground state and phonon excitation modes. The inset demonstrates the optimal adsorption active sites of the intermediates on the Janus monolayers. (c) The orbital contribution of pCOHP between intermediates (OH^*^) on the WSSe with the ground state and *A*2 1 excitation mode. The *p*‐orbital projected density of states of (d,e) Janus MoSSe and (f,g) WSSe monolayers under (d,f) ground state and (e,g) phonon excitation modes. The Fermi level is set as 0 eV. The shaded area corresponds to the filled states up to the Fermi level. The red and gray lines illustrate the −|*ε*|, which is obtained from the structures shown in Figure 2e,f.

The electronic energy plays a crucial role in the OER of Janus MoSSe or WSSe monolayers. Exploring the electronic structure of defective MoSSe and WSSe monolayers in the ground state and excited state is essential to understanding the mechanism of phonon engineering‐induced enhancement of the OER. It is recognized that the S (or Se) *p* orbitals near the Fermi level play a decisive role in mediating interactions with OER intermediates. Accordingly, the density of states (DOS) of atoms located at the catalytic sites in MoSSe and WSSe excited by different lattice vibration modes is examined, where O and H species directly bond with the S or Se atoms, as demonstrated in Figure 5c–f, emphasizing the critical role of the *p* orbitals of S and Se atoms for the OH^*^ intermediate. The OER activity of MoSSe and WSSe monolayers in their excited states can be rationalized through projection band center (*ε*) analysis, as the formation of Se─H and S─H bonds is predominantly governed by the *p* orbitals of S or Se atoms at the catalytic sites, which can be calculated by Equation S24. The obtained *p*‐band center of the observed system is addressed between −1 and 0 eV, which is calculated as Figure 5c–f. One can see that the formation of OH^*^ during the OER is primarily governed by the *p* orbitals of Se (MoSSe) or S (WSSe) atoms at the active sites. This demonstrates that the states near the Fermi level for the *p* orbitals play a crucial role. The stronger adsorption can be induced by −|*ε*| approaching the Fermi level. Thus, the *A*1 1 and *A*2 1 modes can stimulate the outstanding OER activity of MoSSe and WSSe monolayers, which is attributed to the favorable positioning of the *p* band center. Besides, the PDOS of the MoSSe and WSSe monolayers under other phonon excitation modes are demonstrated in Figure S7a–f.

To further understand how the lattice vibrations influence the interaction between the intermediates of the rate‐determining step in the OER of the WSSe monolayer, showing the most pronounced phonon dependence (*A*2 1 mode), the projected crystal orbital Hamilton populations (pCOHP) are also calculated to explore the mechanism of the enhanced OER performance. The rate‐determining step of the WSSe monolayer in the OER presents a pronounced improvement effect; thus, the intermediates (OH^*^) on the WSSe are selected, and the pCOHP of the H─S and O─S interactions is obtained as Figure 5g. Since the bonded state unoccupancy demonstrates the interaction between H (or O) and S atoms, the more negative integrated COHP (ICOHP) value for H─S (−0.11655) and O─S (−0.04602) in WSSe system at the ground state presents a stronger bonding interaction, comparing with that of H─S (−0.00098) and O─S (−0.01769) in *A*2 1mode excited WSSe system, thereby suggesting the decreased overpotential of the rate‐determining step in the later system.

It should be emphasized that the *A*1 1 and *A*2 1mode excited structure of MoSSe and WSSe are not arbitrarily optimized configurations, but are obtained through targeted excitation of specific intrinsic phonon modes, which can induce directional lattice vibrations in the MoSSe and WSSe monolayers, respectively. For the MoSSe system, the *A*1 1 phonon‐driven lattice distortion leads to a noticeable shortening of the distance between the Mo active site and the adsorbed ^*^OH intermediate at the rate‐determining step. Comparing with the orbital hybridization of the ground‐state MoSSe, shown in Figure 6a, this geometric proximity directly enhances the overlap between Mo‐*d* orbitals and O‐*p* states, as reflected by the increased *d*–*p* hybridization near the Fermi level in the PDOS (Figure 6b). The strengthened orbital coupling also facilitates more efficient charge transfer (detail as Equation S25) from Mo to the oxygen‐containing intermediate, stabilizing the reaction intermediate and reducing the kinetic barrier of the rate‐determining step, shown as insets of the Figure 6a,b. Similarly, in the WSSe system, the targeted *A*2 1 phonon excitation drives the W center closer to the ^*^OH intermediate, resulting in a more intimate W─O interaction. Compared with orbit hybridization of the ground state in Figure 6c, the reduced W─OH distance effectively amplifies the hybridization between W‐*d* and O‐*p* orbitals calculated in Figure 6d, as evidenced by the enhanced orbital overlap around the Fermi level and the increased electron transfer to ^*^OH. This phonon‐induced electronic reconfiguration optimizes the adsorption strength of the critical OER intermediate and promotes faster reaction kinetics. Overall, these results demonstrate that selective phonon excitation serves as an internal driving force to dynamically modulate the metal–intermediate distance, thereby tuning orbital hybridization at the active site. By enhancing the *d*–*p* coupling between Mo or W centers and oxygenated intermediates at the rate‐determining step, the phonon‐engineered structures exhibit lowered reaction barriers and superior OER performance of the MoSSe and WSSe monolayers. This mechanism highlights a phonon‐mediated strategy for catalytic optimization that goes beyond static structural or compositional design.

**FIGURE 6:**
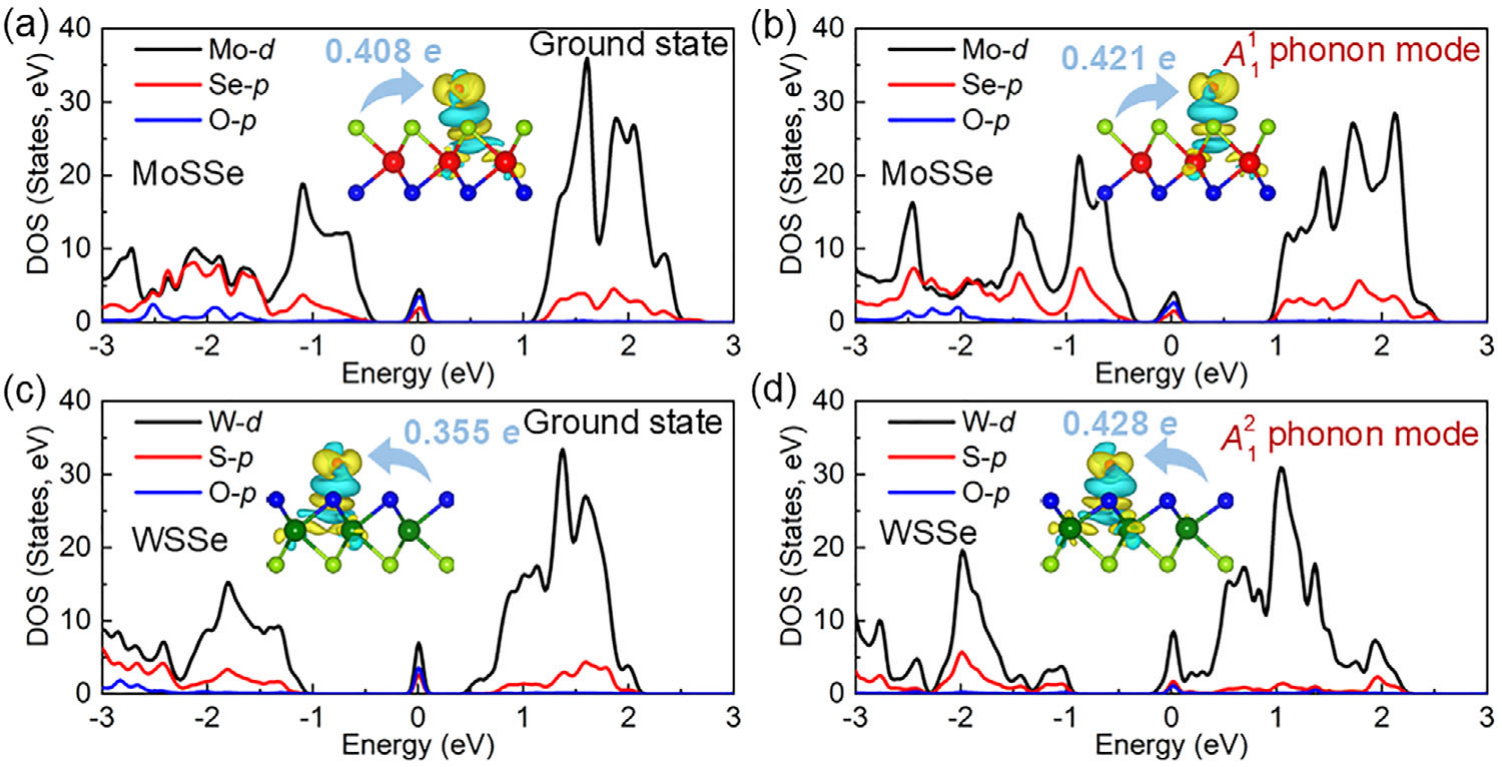
The projected density of states (PDOS) of the ^*^OH complexes at the rate‐determining step of the OER for (a,b) MoSSe and (c,d) WSSe systems under (a,c) ground state and (b) phonon excitation modes. The insets represent the charge density difference of the Janus systems and are visualized using an isosurface level of 0.0002 |*e*|. Regions of electron accumulation and depletion are denoted by yellow and blue, respectively.

Moreover, it should be noted that catalytic activity is generally influenced by multiple descriptors, including electrical conductivity [53], density of active sites [54], surface area [55], and surface reconstruction [56], etc. In practical catalytic systems, these factors are often strongly coupled, making it difficult to quantitatively separate their individual contributions. In the present work, however, the catalyst composition, surface morphology, and active‐site distribution were intentionally kept identical. Consequently, surface area, active‐site density, and reconstruction effects remain unchanged, and the only variable introduced is the phonon‐induced lattice distortion. Therefore, the observed enhancement in OER activity can be directly attributed to phonon‐mediated electronic reconstruction, including the modulation of orbital hybridization, *p*‐band center position, and adsorption energetics of reaction intermediates. We therefore regard phonon engineering not as a replacement for conventional catalyst optimization strategies, but as an additional dynamic degree of freedom that can operate synergistically with conductivity engineering, defect engineering, and surface reconstruction.

Furthermore, it should be noted that the high‐frequency optical phonons investigated in this work are not assumed to be thermally populated under equilibrium conditions. According to the Bose–Einstein distribution, the occupation number of optical phonons with frequencies in the range of 10–15 THz remains relatively low at room temperature [44]. However, infrared‐active optical phonons can be coherently excited to large amplitudes using resonant terahertz or mid‐infrared laser pulses, producing transient lattice distortions far beyond equilibrium thermal fluctuations [43]. In particular, the *A*2 1 mode of Janus WSSe is infrared active and involves out‐of‐plane atomic vibrations that strongly couple to the electronic states near the band edges. Such modes can be selectively driven by intense THz fields through resonant phonon pumping, enabling nonequilibrium phonon populations and transient modification of catalytic active sites [57, 58]. Therefore, the frozen‐phonon configurations considered here should be viewed as representative snapshots of coherently excited phonon states rather than thermally activated lattice vibrations. The calculated catalytic enhancement thus reflects the intrinsic capability of mode‐selective phonon excitation to regulate catalytic energetics.

Although the equilibrium occupation of the *A*2 1 optical phonon is limited at ambient temperature, coherent phonon generation techniques provide a practical route to access such vibrational states experimentally [59]. Recent ultrafast spectroscopy studies have shown that resonantly excited optical phonons can survive over several picoseconds and induce substantial transient modifications in electronic structures, phase transitions, and carrier transport properties [21]. Since catalytic elementary steps typically occur on much longer timescales, repeated optical pumping can continuously maintain nonequilibrium phonon populations and dynamically modulate adsorption energetics. Therefore, the present mechanism is expected to be experimentally accessible using operando terahertz or mid‐infrared excitation, where periodic coherent phonon pumping continuously maintains nonequilibrium lattice vibrations during catalytic operation. Such experimental schemes have recently become feasible with advances in ultrafast pump–probe spectroscopy and nonlinear phononics.

It should be noted that transition‐metal sulfides commonly undergo surface reconstruction under anodic OER conditions, forming metal oxyhydroxide phases that are often regarded as the actual catalytically active species [44, 56, 60]. In our work, however, the objective is not to identify the actual catalytically active phase under realistic operating conditions, but rather to establish the fundamental mechanism by which intrinsic phonon excitation modulates the electronic structure and adsorption energetics. Therefore, pristine Janus TMD monolayers were intentionally adopted as well‐defined model systems to isolate the phonon‐induced effects without the additional complexity introduced by electrochemical reconstruction. We acknowledge that incorporating operando surface reconstruction into phonon‐resolved calculations represents an important and interesting direction for future investigation.

## 4 Conclusions

In summary, we demonstrate that intrinsic lattice vibrations can actively modulate both the electronic structure and catalytic performance of transition metal dichalcogenides. First‐principles calculations reveal that mode‐selective phonon excitations dynamically tune band structures and systematically lower the OER overpotential. In pristine MoS_2_ and WS_2_ monolayers, specific phonon modes reduce the rate‐determining step by up to 7% and 5%, respectively. In Janus systems, phonon symmetry plays a decisive role: degenerate modes preserve the direct bandgap, whereas non‐degenerate excitations induce a direct–indirect transition. Notably, excitation of the *A*1 2 mode in WSSe at ∼402.87 cm^−1^ leads to pronounced bandgap renormalization to 0.93 eV, accompanied by a substantial enhancement in catalytic activity. Mechanistic analysis based on crystal orbital Hamilton population and *p*‐band center descriptors reveals that phonon‐driven lattice displacements weaken the adsorption strength of key reaction intermediates, thereby accelerating catalytic kinetics. These findings establish intrinsic phonon modes as an active and tunable degree of freedom for controlling catalytic energetics. More broadly, this work introduces a dynamic paradigm for catalyst design, in which lattice vibrations, rather than static structure alone, govern reactivity, opening new avenues for phonon‐engineered electrocatalysis across 2D materials.

## Author Contributions

**Yong‐Wei Zhang**: writing – review and editing, investigation. **Kai Ren**: writing – review and editing, writing – original draft. **Liangzhi Kou**: writing – review and editing, investigation, supervision. **Tianyang Liu**: resources, project administration. **Yu Jing**: funding acquisition, writing – review and editing, software, supervision. **Feifan Wang**: methodology, data curation.

## Conflicts of Interest

The authors declare no conflicts of interest.

## Supporting information




**Supporting File**: advs76843‐sup‐0001‐SuppMat.pdf.

## Data Availability

The data that support the findings of this study are available from the corresponding author upon reasonable request.
