# Supplementary material for: Intrinsic Phonons as a Dynamic Control Knob for Catalytic Reactivity in 2D Materials
Source: Adv Sci (Weinh). 2026 Jul 27:e76843. Online ahead of print. doi: 10.1002/advs.76843 (PMC13403750; doi:10.1002/advs.76843)
Supplement: Supplementary file 1 — Supporting File: advs76843‐sup‐0001‐SuppMat.pdf. [file ADVS-9999-e76843-s001.pdf]

Supporting Information

Intrinsic Phonons as a Dynamic Control Knob for Catalytic Reactivity in 2D Materials

Kai Ren[1,#], Feifan Wang[2,#], Yong-Wei Zhang[3], Tianyang Liu[2], Liangzhi Kou[4,*] and Yu Jing[2,*]

[1]School of Mechanical and Electronic Engineering, Nanjing Forestry University, Nanjing 210037, China

[2]Co-Innovation Centre of Efficient Processing and Utilization of Forest Resources, College of Chemical Engineering, Nanjing Forestry University, Nanjing 210037, China

[3]Institute of High Performance Computing, A*STAR, Singapore 138632, Singapore

[4]School of Mechanical, Medical and Process Engineering, Queensland University of Technology, Gardens Point Campus QLD 4001, Brisbane, Australia

Corresponding author: liangzhi.kou@qut.edu.au (L. K), yujing@njfu.edu.cn (Y. J)

K. R and F. W contributed equally to this work.

The potential energy of the TMDs crystal can be expressed as a power expansion in terms of the nuclear displacements (u):

$$\phi = \phi_0 + \phi_1 + + \cdots \tag{S1}$$

where ϕ_1 and ϕ_2 can be obtained as:

$$\phi_1 = \sum_{l\kappa x} \frac{\partial \phi}{\partial x(l\kappa)} u_x(l\kappa), \tag{S2}$$

$$\phi_2 = \frac{1}{2} \sum_{l\kappa x} \sum_{l'\kappa' y} \frac{\partial^2 \phi}{\partial x(l\kappa) \partial y(l'\kappa')} u_x(l\kappa) u_y(l'\kappa'). \tag{S3}$$

Table S1. The calculated displacement of lattice vibrations of Mo, S, Se, and W atoms in MoSSe monolayer at different intrinsic phonon excited state.

Vibration frequency	Atoms	x	y	z
E^1	Mo	0.039563	0.00	0.00
202.64 cm^{-1}	S	0.092171	0.00	0.00

	Se	−0.085513	0.00	0.00
A_1^1	Mo	0.00	0.00	−0.046413
286.47 cm^{-1}	S	0.00	0.00	−0.076642
	Se	0.00	0.00	0.087529
E^2	Mo	0.003372	−0.063348	0.00
347.91 cm^{-1}	S	−0.007104	0.133452	0.00
	Se	−0.001214	0.022795	0.00
A_1^2	Mo	0.00	0.00	0.058614
437.84 cm^{-1}	S	0.00	0.00	−0.143113
	Se	0.00	0.00	−0.013116

Table S2. The calculated displacement of lattice vibrations of W, S, Se, and W atoms in WSSe monolayer at different intrinsic phonon excited state.

Vibration frequency	Atoms	*x*	*y*	*z*
E^1	W	0.029678	−0.000579	0.00
197.21 cm^{-1}	S	0.063252	−0.001234	0.00
	Se	−0.094786	0.001850	0.00
A_1^1	W	0.00	0.00	−0.033916
274.73 cm^{-1}	S	0.00	0.00	−0.042461
	Se	0.00	0.00	0.096200
E^2	W	0.000249	−0.034159	0.00
320.57 cm^{-1}	S	−0.001125	0.154246	0.00
	Se	−0.000123	0.016897	0.00
A_1^2	W	0.00	0.00	0.029966
402.87 cm^{-1}	S	0.00	0.00	−0.161223
	Se	0.00	0.00	−0.004301

When we include a phonon degree of freedom (frequency, ω_{ph}) into the Hamiltonian of the MoSSe and WSSe systems, the form of the Schrödinger equation itself does not change, which is still expressed by Eq. (S4) in supporting Information.

$$i\hbar\partial\Psi(t)/\partial t=\hat{H}\Psi(t), \quad \text{(S4)}$$

while the Hamiltonian ($\hat{H}$) should consider the extra phonon operators and possibly electron–phonon coupling terms.

The long-wavelength optical phonon modes of the MoS_2 or WS_2 at the Γ point can be decomposed as follows:

$$\Gamma_{\text{optical}}(MoS_2 \text{ or } WS_2)=E^2\,(\text{R})+E^1\,(\text{R})+A_1^1\,(\text{IR}+\text{R})+A_2^2\,(\text{IR}), \quad \text{(S5)}$$

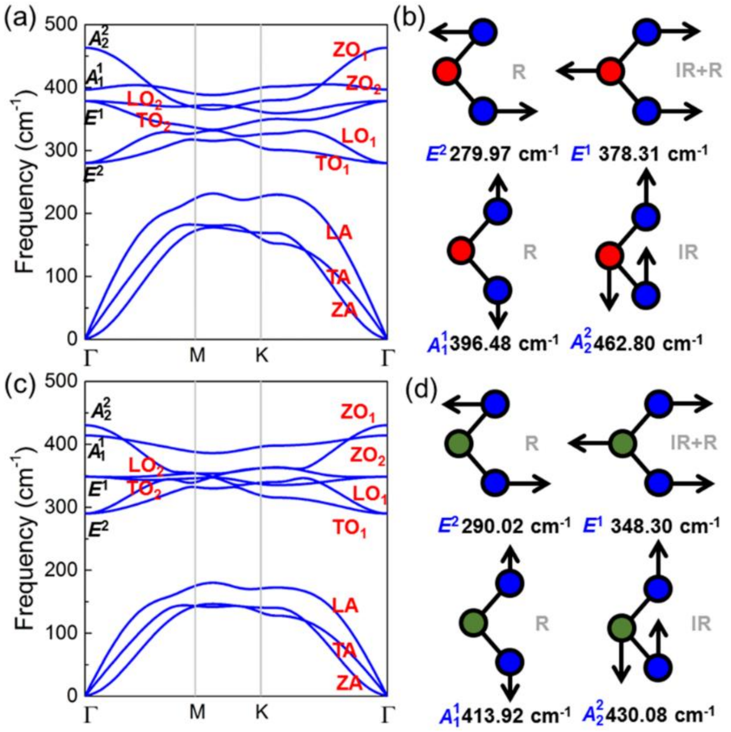

Figure S1. (a, c) The phonon scattering spectrum and the (b, d) lattice vibration modes of (a, b) MoS_2 and (c, d) WS_2 monolayers. Blue and green spheres represent the S and Mo atoms, respectively.

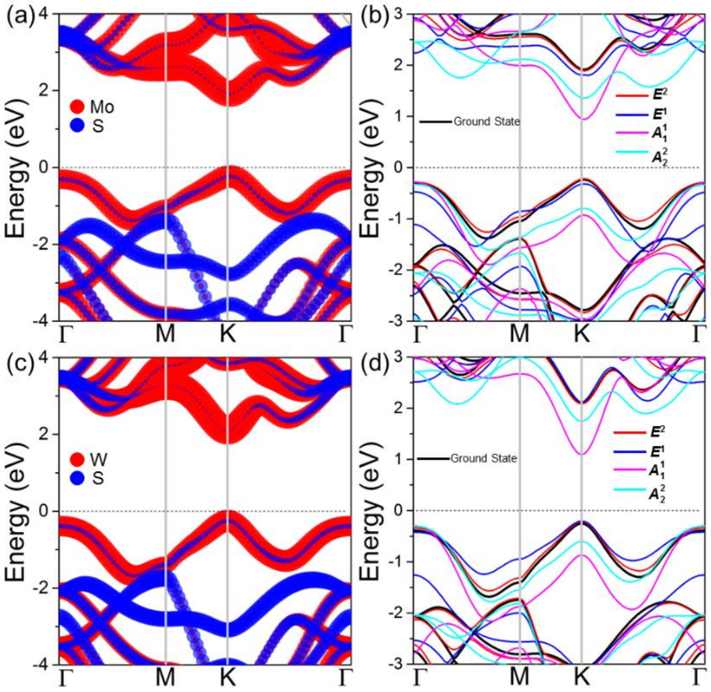

Figure S2. The projected band structure of the (a) MoS_2 and (c) WS_2 monolayers with ground state. The tunable band structures of the (b) MoS_2 and (d) WS_2 monolayers under different lattice vibration modes. The Fermi level is marked as 0 eV.

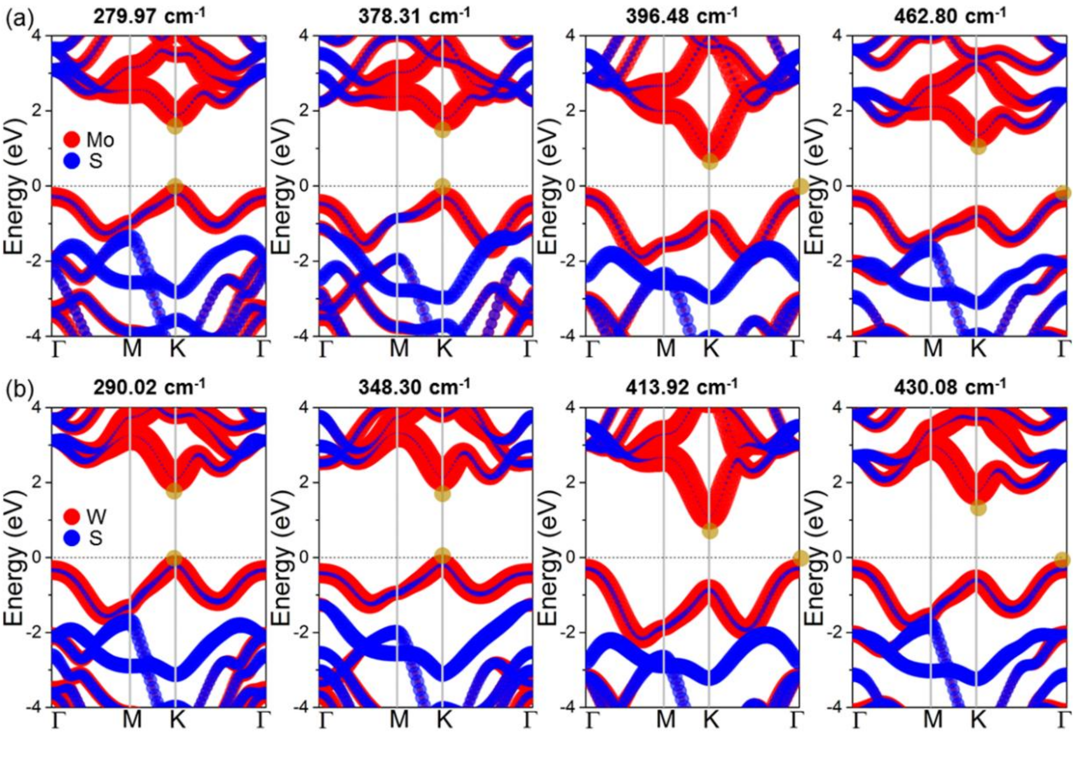

Figure S3. The projected band structure of the (a) MoS_2 and (b) WS_2 monolayers excited by the lattice vibration modes with different phonon frequency at Γ point.

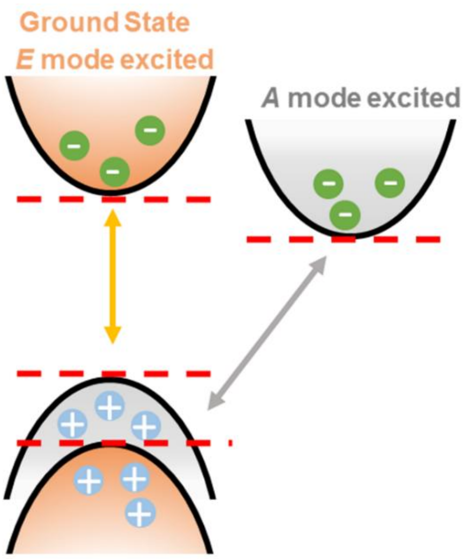

Figure S4. The band edge positions of the MoS_2 or WS_2 monolayer excited by the lattice vibration mode.

The light absorption capacity of the MoSSe and WSSe can be calculated by the light absorption coefficient:[1]

$$\alpha(\omega)=\frac{\sqrt{2}\omega}{c}\left\{\left[\varepsilon_1^2(\omega)+\varepsilon_2^2(\omega)\right]^{1/2}-\varepsilon_1(\omega)\right\}^{1/2}. \tag{S6}$$

where real and imaginary parts for the dielectric constant are expressed by the $\varepsilon_1(\omega)$ and $\varepsilon_2(\omega)$, respectively. Furthermore, ω, α and c represent the angular frequency, the absorption coefficient and speed of light in vacuum, respectively. Besides, $\varepsilon_2(\omega)$ can be calculated as:[2]

$$\varepsilon_2\left(q\to O_{\hat{u}},\hbar\omega\right)=\frac{2e^2\pi}{\Omega\varepsilon_0}\sum_{k,v,c}\left|\langle\Psi_k^c\,|\,\hat{u}\cdot r\,|\,\Psi_k^v\rangle\right|^2\times\delta\left(E_k^c-E_k^v-E\right), \tag{S7}$$

where ψ_k represents the wave function, E_k is the energy and $\hat{u}$ is the unit vector of the electric field of the incident light. The conduction bands and valence bands are expressed by superscripts (v and c) in ψ_k, E_k, respectively. The complex dielectric function is $\varepsilon(\omega)=\varepsilon_1(\omega)+i\varepsilon_2(\omega)$, and the real part ε_1 can be obtained from ε_2 by using the Kramers–Kronig relation.[3]

The overall OER of the TMDs monolayer can be expressed by:[4]

$$2H_2O(l) \rightarrow O_2(g) + 4H^+ + 4e^-. \quad (S8)$$

The overall reaction demonstrates four elementary reaction steps, involving the transfer of one electron, which can be further explained as:[5]

$$H_2O(l) + * \rightarrow OH* + H^+ + e^-, \quad (S9)$$

$$OH* \rightarrow O* + H^+ + e^-, \quad (S10)$$

$$O* + H_2O\ (l) \rightarrow OOH* + H^+ + e^-, \quad (S11)$$

$$OOH* \rightarrow OO* + H^+ + e^-, \quad (S12)$$

$$OO* \rightarrow * + O_2(g) + H^+ + e^-, \quad (S13)$$

where * expresses the active site on the MoSSe or WSSe monolayer. $H_2O(l)$ and $O_2(g)$ demonstrate a liquid-phase water molecule and a gas-phase O_2 molecule, respectively. The O*, OH*, OO* and OOH* are used to suggest the adsorbed intermediates in the OER. H^+ is a proton, while the e^- represents an electron. The binding energies of different adsorbed system for the intermediates is calculated as:[6]

$$\Delta E_{OH*} = E_{TMDs+OH*} - E_{TMDs} - (E_{H2O} - 1/2E_{H2}), \quad (S14)$$

$$\Delta E_{O*} = E_{TMDs+O*} - E_{TMDs} - (E_{H2O} - E_{H2}), \quad (S15)$$

$$\Delta E_{OOH*} = E_{TMDs+OOH*} - E_{TMDs} - (2E_{H2O} - 3/2E_{H2}), \quad (S16)$$

$$\Delta E_{OH*} = E_{TMDs+OH*} - E_{TMDs} - (E_{H2O} - 1/2E_{H2}), \quad (S17)$$

$$\Delta E_{OO*} = E_{TMDs+OO*} - E_{TMDs} - (2E_{H2O} - 2E_{H2}), \quad (S18)$$

where the ΔE_{OH*}, ΔE_{O*}, ΔE_{OOH*} and ΔE_{OO*} represent the energy of the intermediates. E_{TMDs} suggests the pure MoSSe or WSSe monolayer. Besides, the $\Delta E_{TMDs+O*}$, $\Delta E_{TMDs+OOH*}$, $\Delta E_{Janus+OO*}$ and $\Delta E_{TMDs+OH*}$ represent the binding energy of the intermediates absorbed Janus TMDs system. E_{H2O} and E_{H2} is the energy of the water and H_2 molecules, respectively. In addition, the Gibbs free energy difference of the different OER is calculated as:

$$\Delta G_1 = G_{OH*}, \quad (S19)$$

$$\Delta G_2 = G_{O*} - G_{OH*}, \quad (S20)$$

$$\Delta G_3 = G_{OOH*} - G_{O*}, \quad (S21)$$

$$\Delta G_4 = G_{OO*} - G_{OOH*}, \quad (S22)$$

$$\Delta G_5 = 4.92 - G_{OO*}, \quad (S23)$$

where the total difference of the Gibbs free is 4.92 eV used in overall $2H_2O \rightarrow O_2 + 2H_2$ at 298.15 K.

The OER activity of Janus TMDs monolayers in their excited states can be rationalized through projection band center (ε) analysis, as the formation of Se–H and S–H bonds is predominantly governed by the p orbitals of S or Se atoms at the catalytic sites, which can be calculated by:

$$\varepsilon = \frac{\int_{-\infty}^{\infty} x\rho(x)dx}{\int_{-\infty}^{\infty} \rho(x)dx}. \tag{S24}$$

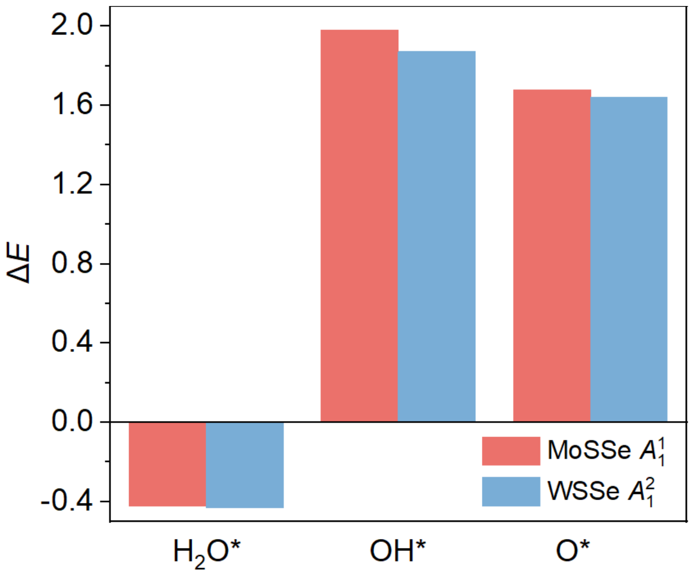

Figure S5. The calculated adsorption energy of the H_2O*, OH* and O* on the structures of the A_1^1 mode of MoSSe and the A_1^2 mode of WSSe systems.

Obviously, the reaction of O* to OOH* decides the overpotential of the rate-determining step of MoS_2 and WS_2 monolayers in the OER by 2.272 eV and 1.963 eV, respectively, under the ground state. It is worth noting that, the OER Gibbs free energy of MoS_2 and WS_2 monolayers under phonon intrinsic excited states is mainly addressed in the O* and OOH* processes, shown as Figure S5a and b, and the rate-determining step still retains as third step excited by different intrinsic lattice vibration. However, the overpotential of the OER is reduced as 2.108 eV and 1.868 eV for MoS_2 and WS_2

monolayers, respectively, which means the A_2^2 mode can decrease the overpotential of the ground state by 7% and 5%, respectively.

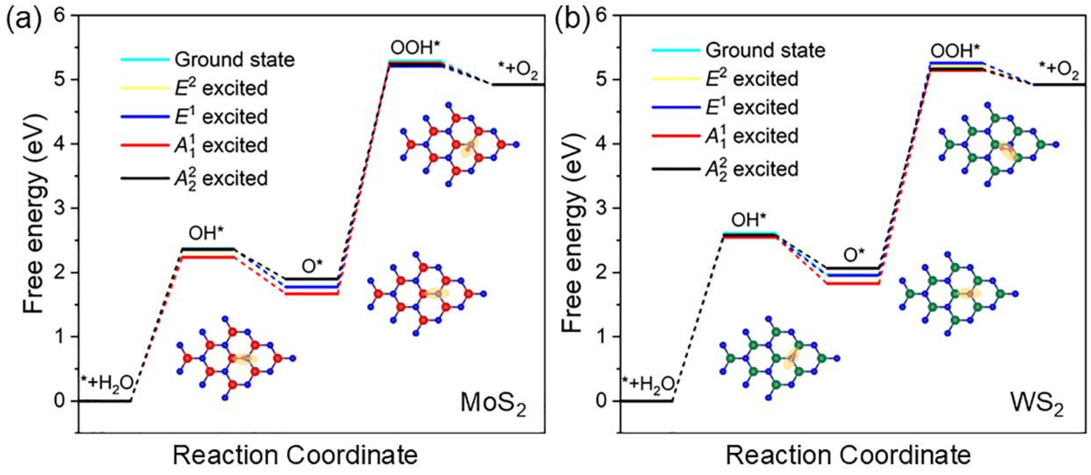

Figure S6. The calculated Gibbs free energy of the adsorbed intermediates in the OER on (a) MoS_2 and (b) WS_2 monolayers with the ground state and phonon excitation modes. The inset demonstrates the optimal adsorption active sites of the OH*, O* and OOH* intermediates on the systems.

The charge difference ($\Delta\rho$) in the Janus TMDs and intermediates is calculated by Bader charge analysis,[7-9] which can be calculated using the following equation:

$$\Delta\rho = \rho_{\mathrm{sys}} - \rho_{\mathrm{Janus}} - \rho_{\mathrm{intermediates}}, \tag{S25}$$

where ρ_{sys}, ρ_{Janus} and $\rho_{\mathrm{intermediates}}$ are the total charge density of adsorbed system, pristine Janus TMDs and intermediates, respectively.

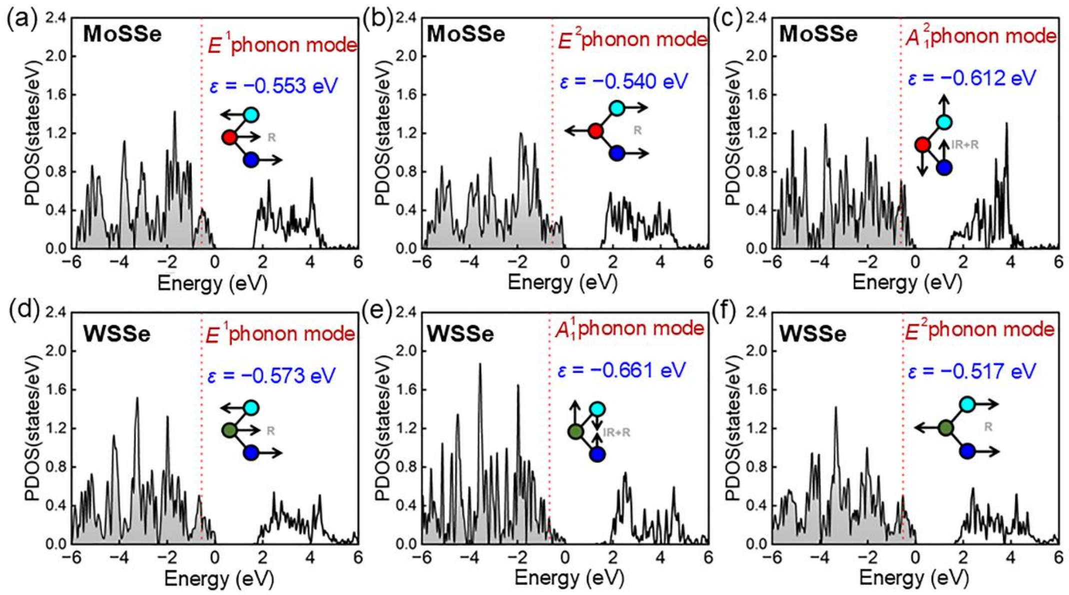

Figure S7. The *p*-orbital projected density of states of (a, b, c) Janus MoSSe and (d, e, f) WSSe monolayers under (a, d) E^1, (b, f) E^2, (c) A_1^2 and (e) A_1^1 phonon excitation modes. The Fermi level is set as 0 eV. The shaded area corresponds to the filled states up to the Fermi level. The red lines illustrate the $-|\varepsilon|$, which is obtained by the structures shown in Figure 3 (e) and (f).

Reference

1. J. Liao, B. Sa, J. Zhou, R. Ahuja and Z. Sun, *J. Phys. Chem. C*, 2014, **118**, 17594−17599.
2. G. Zhang, M.-B. Yu, C.-H. Tung and G.-Q. Lo, *IEEE Electron Device Lett.*, 2008, **29**, 1302−1305.
3. A. Ashoka, R. R. Tamming, A. V. Girija, H. Bretscher, S. D. Verma, S.-D. Yang, C.-H. Lu, J. M. Hodgkiss, D. Ritchie and C. Chen, *Nature communications*, 2022, **13**, 1437.
4. K. Ren, K. Wang, Y. Cheng, W. Tang and G. Zhang, *Nano Futures*, 2020, **4**, 032006.
5. M. Pu, Y. Guo and W. Guo, *Nanoscale Horiz*, 2022, **7**, 1404−1410.
6. M. Pu, Y. Guo and W. Guo, *Nanoscale*, 2021, **13**, 20576−20582.
7. G. Henkelman, A. Arnaldsson and H. Jónsson, *Comp. Mater. Sci*, 2006, **36**, 354−360.
8. W. Tang, E. Sanville and G. Henkelman, *Journal of physics. Condensed matter : an Institute of Physics journal*, 2009, **21**, 084204.

9. E. Sanville, S. D. Kenny, R. Smith and G. Henkelman, *Journal of computational chemistry*, 2007, **28**, 899−908.
